# How to preserve and handle fish liver samples to conserve RNA integrity

**DOI:** 10.1007/s11356-019-05033-0

**Published:** 2019-04-22

**Authors:** Eeva-Riikka Vehniäinen, Maiju Ruusunen, Pekka J. Vuorinen, Marja Keinänen, Aimo O. J. Oikari, Jussi V. K. Kukkonen

**Affiliations:** 1 Department of Biological and Environmental Science, University of Jyväskylä, P.O. Box 35, FI-40014 Jyväskylän yliopisto, Finland; 2 Natural Resources Institute Finland (Luke), P.O. Box 2, FI-00791 Helsinki, Finland

**Keywords:** Bleached kraft pulp mill effluent, Cytochrome p450, Liver, Quantitative reverse transcription PCR, RNA integrity, Sample storage

## Abstract

As transcriptomic studies are becoming more and more common, it is important to ensure that the RNA used in the analyses is of good quality. The RNA integrity may be compromised by storage temperature or freeze-thaw cycles, but these have not been well studied in poikilothermic fishes. This work studied the effects of tissue storage time and temperature, and freeze-thaw cycles of tissue and extracted RNA on RNA integrity in brown trout (*Salmo trutta* L.) liver. The storage time and temperature had an effect on RNA integrity, but RNA suitable for quantitative reverse transcription PCR (RT-qPCR) (RIN > 7) was still obtained from samples preserved at − 20 °C for 6 months. Freeze-thaw cycles of tissue or RNA did not compromise the integrity of RNA. RNA degradation had an effect on RT-qPCR results, and the effect depended on gene. The RT-qPCR analysis of historical samples from a bleached kraft pulp mill effluent exposure in 1984 revealed no significant *cyp1a* induction. Recommendations are given for the preservation and handling procedures of samples designated for transcriptomic analyses.

## Introduction

The studies using transcriptomics are becoming more and more common in ecotoxicology. To make sure that the results are trustworthy, it is absolutely crucial to use good-quality RNA in the analyses. The RNA is considered fragile, and the recommendation is to flash-freeze the samples in liquid nitrogen, to preserve them at − 70 °C or lower, and to avoid freeze-thaw cycles both of the tissue sample and of the extracted RNA (Sambrook and Russell 2001). However, in field conditions these procedures are not always possible, as liquid nitrogen, − 70 °C freezer, or products like RNA later may not be available. The freeze-thaw cycles of the frozen sample or the extracted RNA can be avoided by taking subsamples at the time of sampling or RNA extraction. Sometimes, the need for multiple analyses arises only after the sample or RNA has already been preserved in one tube, which necessitates multiple thawing. It is therefore important to know how sample preservation and repetitive thawing affect the integrity of the RNA.

The RNA integrity can be assessed most reliably with microchip gel electrophoresis, using Agilent BioAnalyzer or Bio-Rad Experion (Imbeaud et al. 2005). The BioAnalyzer calculates an RNA integrity number (RIN) for each sample using the electrophoresis results (Schroeder et al. 2006). The RIN is a measure of how intact the RNA is, and it ranges from 1 (totally degraded RNA) to 10 (fully intact RNA) (Schroeder et al. 2006). Several RIN cut-offs ranging from 3.95 to 8 have been proposed for transcriptomic analyses (Fleige and Pfaffl 2006; Gallego Romero et al. 2014; Huang et al. 2013; Ibberson et al. 2009; Imbeaud et al. 2005; Weis et al. 2007).

Studies on the effects of sample handling and preservation on RNA integrity have mainly been conducted on mammalian species, having a normal body temperature ca. 15 °C higher than room temperature (Bao et al. 2013; Ervin et al. 2007; Micke et al. 2006; Ohashi et al. 2004; Thompson et al. 2007; Viana et al. 2013; Walter et al. 2006). Poikilothermic cold-water fish thrive in temperatures from near zero close to 20 °C. The only study on fish investigated the effect of room temperature incubation on the quality of RNA in Atlantic salmon (*Salmo salar* L.) tissues and found that the effect depended on tissue, liver being the tissue with very rapid RNA degradation (Seear and Sweeney 2008). The effects of freeze-thaw cycles of tissue and RNA have only been studied in mammals (Botling et al. 2009; Jochumsen et al. 2007; Thompson et al. 2007), and no published studies exist about storing the samples at − 20 °C.

The aim of this study was to investigate how tissue handling, storage time, storage temperature, and freeze-thaw cycles affect the quality of RNA of fish liver. Liver tissue of brown trout (*S. trutta* L.) was preserved at room temperature, fridge, normal freezer, deep freezer, or liquid nitrogen for various time periods up to 6 months. In addition, the effect of freeze-thaw cycles on RNA integrity was studied by analyzing RIN after repetitive thawing and freezing liver tissue and purified RNA. The effect of RNA integrity on RT-qPCR results was investigated by room temperature incubation of liver samples, resulting in RNA with varying RINs. RNA was also extracted from historical samples that had been stored for 28 years in liquid nitrogen, and RT-qPCR run for them.

## Material and methods

### Tissue sampling and storage

**Fig. 1:**
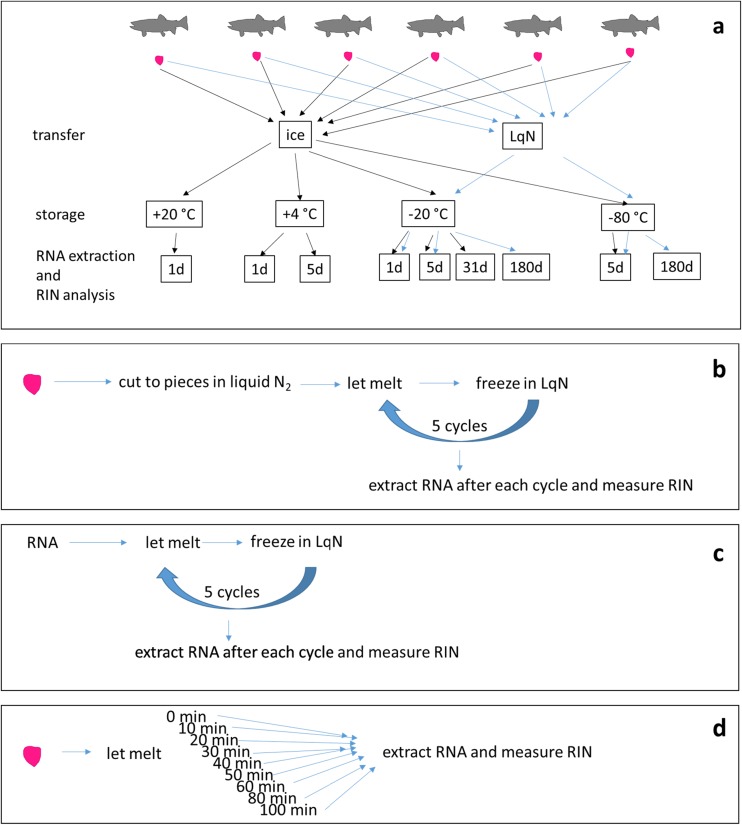
Experimental plan of the study. Livers of six brown trout (*Salmo trutta*) were dissected, transferred in two conditions, and stored at four temperatures before RNA extraction and RNA integrity (RIN) analysis (**a**). Black arrows refer to samples transported on ice, blue arrows to those transported in liquid nitrogen (LqN). Other experiments included freeze-thaw cycles of liver (**b**) and RNA (**c**) and incubation of the frozen liver in room temperature for nine periods of time (**d**) before RNA extraction and RIN analysis

The new liver tissue samples were taken from six four-year-old male brown trout (*Salmo trutta L.*) with the mean body weight of 1006 g in Laukaa fish farm of the Natural Resources Institute Finland (Luke, formerly the Finnish Game and Fisheries Research Institute) in November 2012. The brown trout were taken from water of < 5 °C and the sampling was performed in a low-temperature room. The fish were stunned with a blow to the head, weighed, and their length was measured, after which they were sacrificed by nuchal break. Livers were removed and cut to 50–100 mg pieces randomly, avoiding connective tissue areas. They were placed in microcentrifuge tubes, and either immediately frozen in liquid nitrogen or kept on ice. In addition, larger liver samples of up to 1.5 g were taken, and immediately frozen and preserved in liquid nitrogen. The samples were transferred to the University of Jyväskylä (4 h) either in an ice bath or frozen in liquid nitrogen, and then stored at room temperature (+ 20 °C), fridge (+ 4 °C), normal freezer (− 20 °C), or deep freezer (− 80 °C). The RNA extraction was carried out after 1, 5, 31, and 180 (6 months) days of storage. The transfer and storage conditions are summarized in Table 1, and the experimental plan is shown in Fig. 1.

**Table 1:** Transfer (transfer time 4 h from the sampling to the laboratory) and storage conditions of brown trout livers and the respective RNA integrity numbers (RIN). RIN values represent mean ± SD of five (marked with #) or six biological replicates

Transfer	Storage temperature (°C)	Storage time	RIN
Ice	+ 20	1 day	2.8 ± 0.4
Ice	+ 4	1 day	6.8 ± 0.7
Ice	+ 4	5 days	3.7 ± 1.7
Ice	− 20	1 day	9.2 ± 0.3
Ice	− 20	5 days	9.4 ± 0.3
Ice	− 20	31 days	7.6 ± 0.3 #
Ice	− 80	5 days	9.5 ± 0.3
Liquid nitrogen	− 20	1 day	8.0 ± 0.4
Liquid nitrogen	− 20	5 days	8.6 ± 0.8
Liquid nitrogen	− 20	6 months	7.6 ± 0.4 #
Liquid nitrogen	− 80	5 days	9.7 ± 0.3
Liquid nitrogen	− 80	6 months	10 ± 0.0

The historical samples originated from a similar exposure as reported in Vuorinen and Vuorinen (1985), but contrary to the previous study, fish were exposed until they were sacrificed. Briefly, five-year-old brown trout of both sexes (the mean body weight 1061 g) had been exposed to low, sublethal dilutions (0.1, 0.2, and 0.5%) of bleached kraft pulp and paper mill effluents (BKME) or control water for 4 months in July–November 1984. At sampling, the air and water temperatures were < 5 °C. The excised liver samples were immediately frozen in liquid nitrogen. In our study, the RNA was extracted from liver samples that had been preserved in liquid nitrogen for 28 years.

### RNA extraction

The RNA was extracted with Tri reagent (Molecular Research Center) according to the manufacturer’s protocol. Pieces of liver (50–100 mg) were homogenized with plastic homogenization sticks in 1 ml Tri reagent in microcentrifuge tubes. After 5 min incubation at room temperature, 200 μl chloroform was added and the tube was shaken vigorously. The tubes were let stand for 5 to 15 min at room temperature, after which they were centrifuged (12,000 ×*g*, 15 min, + 4 °C). The uppermost supernatant was transferred to a new tube, and the RNA was precipitated by adding 500 μl isopropanol. After centrifugation (12,000×*g*, 8 min, + 4 °C), the precipitate was washed twice with 75% ethanol. The precipitates were stored under 75% ethanol at − 20 °C until processed further. After the ethanol was removed, the precipitates were let dry for 5 to 10 min at room temperature and dissolved in 200 μl of nuclease-free water (Thermo Fisher Scientific Inc.). The dissolved RNAs were stored at − 80 °C.

### Assessment of RNA quality

The concentration (A_260_) and purity (A_260_/A_280_, A_260_/A_230_) of the RNAs were measured with NanoDrop 1000 (Thermo Fisher Scientific). When necessary, the RNAs were diluted to reach the concentration suitable for the RNA integrity analysis (25–500 ng).

The integrity of the RNAs was assessed with microchip electrophoresis using Eukaryote total RNA 6000 nano kit (Agilent) and BioAnalyzer (Agilent) according to the manufacturer’s instructions. The software did not need modification for assessing RINs in brown trout samples.

### Freeze-thaw cycles of liver tissue

Brown trout liver tissue (ca. 1.5 g pieces) stored in liquid nitrogen was cut to 50–100 mg pieces when still frozen (Fig. 1b). The pieces were let to melt in microcentrifuge tubes at room temperature (3 to 5 min) and flash-frozen in liquid nitrogen. When the desired number of freeze-thaw cycles was obtained, the liver tissue was immediately homogenized on ice in Tri reagent and RNA extracted as described above.

### Freeze-thaw cycles of RNAs

The purified RNAs from the liver of five individuals were thawed in total five times, and the integrity of the RNA was assessed after each freeze-thaw cycle (Fig. 1c). The RNAs were thawed at room temperature (ca. 5 min) and transferred on ice immediately after melting. The RNAs remained unfrozen on ice for approximately 30 min. After assessment of RNA integrity, the RNAs were moved back to − 80 °C and kept there for at least 4 h before the next melting.

### Room temperature incubation of liver tissues to obtain RNA with varying RINs

Liver tissue from three BKME-exposed fish that had been stored in liquid nitrogen for 28 years, but still had good RNA quality (RIN > 9.5), was taken to room temperature and let to melt. Pieces of 50 to 100 mg were taken at 0 min (when the tissue was still frozen), and at 10, 20, 30, 40, 50, 60, 80, and 100 min, and the RNA was extracted with Tri reagent as described above.

### Quantitative reverse transcription PCR

Quantitative reverse transcription PCR (RT-qPCR) runs were conducted for all room temperature incubated samples, for three new trout samples, and for historical samples that had a RIN ≥ 6. As the historical pulp and paper mill effluents were known to contain substances that induce its transcription, *cytochrome P450 1a* was chosen as the target gene. The primers were designed with AmplifX (version 1.5.4 by Nicolas Jullien; CNRS, Aix-Marseille Université—http://crn2m.univ-mrs.fr/pub/amplifx-dist) and checked for specificity with Primer-BLAST (http://www.ncbi.nlm.nih.gov/tools/primer-blast/). The features of the primers are shown in Table 2. *60S ribosomal protein l17* (*rl17*) and *nadh dehydrogenase 1 alpha subcomplex subunit 8* (*ndufa8*) were chosen as reference genes because their expression in effluent-exposed and control brown trout was the most stable of a set of reference genes tested (Vehniäinen, unpublished).

**Table 2:** Features of the primers used in the RT-qPCR

Gene name	Accession	Primer sequence	Efficiency	Product length
*60S ribosomal protein l17* (*rl17*)	NM_001195159.1	F: atcgagcacatccaggtcaacaag R: aatgtggcaaggggagctcatgta	100.3	99
*nadh dehydrogenase 1 alpha subcomplex subunit 8* (*ndufa8*)	NM_001160582.1	F: ttcagagcctcatcttgcctgct R: caacatagggattggagagctgtacg	101.1	119
*Cytochrome P450 1 a* (*cyp1a*)	U62796	F: cagtccgccaggctcttatcaagc R: gccaagctcttgccgtcgttgat	102.2	94

After RNA extraction, 1 μg of total RNA was treated with DNase (DNase I, RNase-free; Thermo) according to the manufacturer’s instructions. The RNA was reverse transcribed to cDNA (iScript cDNA Synthesis Kit, Bio-Rad), after which the cDNAs were diluted 1 + 9 with sterile water. Each RT-qPCR reaction consisted of 5 μl of the diluted cDNA, 0.75 μl of forward and reverse primers (final concentration 300 nmol), 6 μl sterile water, and 12.5 μl iQ SYBR Green Supermix (Bio-Rad). The reactions were run in duplicates in clear 96-well PCR plates (Bio-Rad), on a CFX96 Real-Time PCR cycler (Bio-Rad). The protocol was 3 min at 95 °C; 40 cycles (10 s at 95 °C, 10 s at 58 °C, and 30 s at 72 °C); 10 s at 95 °C and melt curve from 65 to 95 °C. No template controls were run for all genes on all plates, and their cycles of threshold (C_T_s) were always over 38. Melt curves showed a single peak, confirming formation of only one PCR product. The expression values were calculated with the 2^−ΔΔ*C*^_T_ method, as the efficiencies of all genes were almost identical and close to 100% (Livak and Schmittgen 2001).

### Statistics

The effect of storage temperature and storage duration on RNA integrity was tested with two-way ANOVA followed by Tukey’s test. The effect of transport temperature on RNA integrity was tested with repeated measures one-way ANOVA with a Greenhouse-Geisser correction for the samples stored at − 20 °C and with *t* test for the samples stored at − 80 °C.

Linear regression curves for C_T_ as a function of sample melting time and RIN were drawn for each gene (*cyp1a*, *ndufa*, and *rl17*), using the data from the room temperature incubated historical liver samples. Analysis of covariance (ANCOVA) was used to compare the linear regressions for C_T_ as a function of sample melting time between different genes (*cyp1a*, *ndufa*, *rl17*) to determine whether they were statistically similar. One-way ANOVA with repeated measures was used to test the effect of melting time on RIN. Because the data violated the assumption of sphericity, a Greenhouse-Geisser correction was used.

Log_10_ transformation was performed for the expression values to meet the requirements of ANOVA (normality, equal variance). One-way ANOVA followed by Tukey’s test was used to test whether the effluent exposure had an effect on the levels of *cyp1a* mRNA. IBM SPSS Statistics 20 was used for the statistical analyses.

## Results

### Storage length and temperature have an effect on RNA integrity

High storage temperatures of liver samples resulted in poor-quality RNA, whereas all storage temperatures below zero yielded RNA with RIN > 7 (Table 1). Even the samples that had been preserved at − 20 °C for 6 months had RINs > 7 and could thus be considered suitable for RT-qPCR (Table 1). Both the length of storage and the storage temperature had an effect on RNA integrity (two-way ANOVA, *p* < 0.001 for both). The RINs were different between all storage temperatures (Tukey, *p* < 0.001 for all pairs). Transfer temperature (ice/liquid nitrogen for 4 h) had an effect on RIN in samples stored at − 20 °C (ANOVA with repeated measures, *p* = 0.05) but not in those stored at − 80 °C (*t* test, *p* > 0.05).

### Freeze-thaw cycles of tissue or RNA do not compromise the integrity of RNA

Freeze-thaw cycles had no effect on RNA integrity. The RNA extracted from liver samples that had been thawed five times was still intact with RIN > 9.5 (Table 3). Similarly, purified RNA remained un-degraded after five freeze-thaw cycles (Table 3).

**Table 3:** The effect of thawing on RIN values in brown trout liver samples and extracted RNA. RIN values represent mean ± SD of five (liver sample) or six (purified RNA) biological replicates

Number of freeze-thaw cycles	Liver sample RIN	Purified RNA RIN
1	9.7 ± 0.2	10.0 ± 0.0
2	9.7 ± 0.1	9.5 ± 0.2
3	9.8 ± 0.3	9.6 ± 0.2
4	9.7 ± 0.2	9.9 ± 0.1
5	9.7 ± 0.2	9.8 ± 0.1

### Room temperature incubation of samples has an effect on RNA integrity and RT-qPCR results

**Fig. 2:**
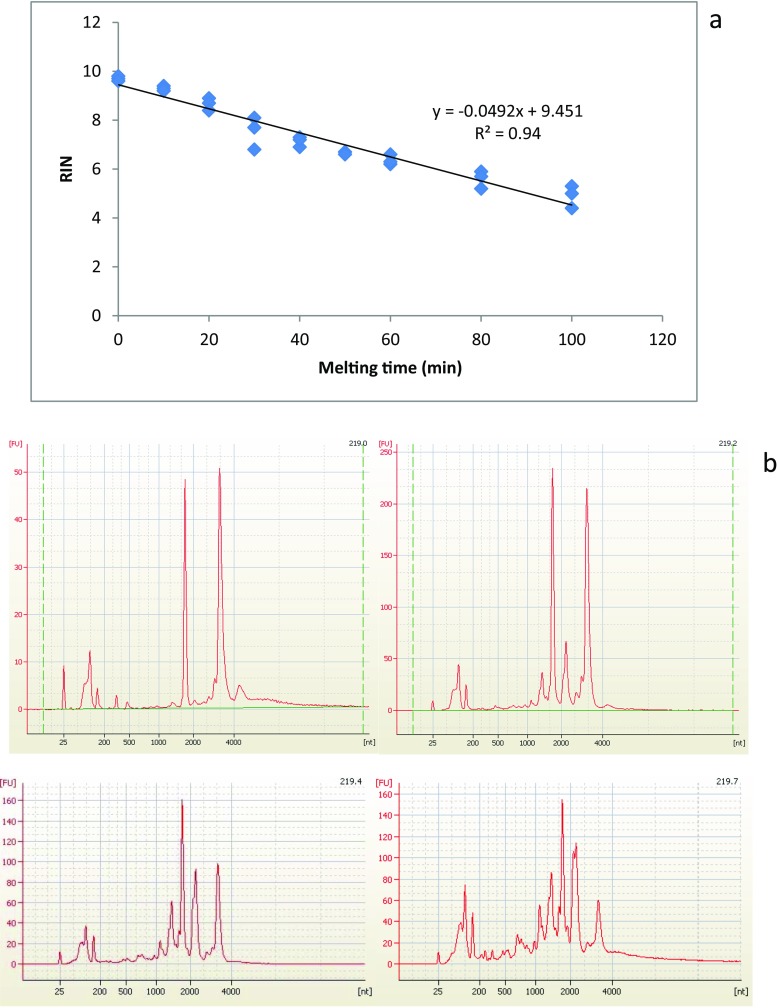
Melting the samples deteriorates the quality of sample RNA in a time-dependent manner. Three brown trout (*Salmo trutta*) livers that had been preserved in liquid nitrogen for 28 years were left to melt at room temperature, and samples collected at 0 to 100 min. RNA was extracted and RIN values assessed. RIN as a function of melting time (**a**). Representative electropherograms (**b**) of RIN 9.7 (219.0), RIN 8.9 (219.2), RIN 6.9 (219.4), and RIN 5.9 (219.7)

**Fig. 3:**
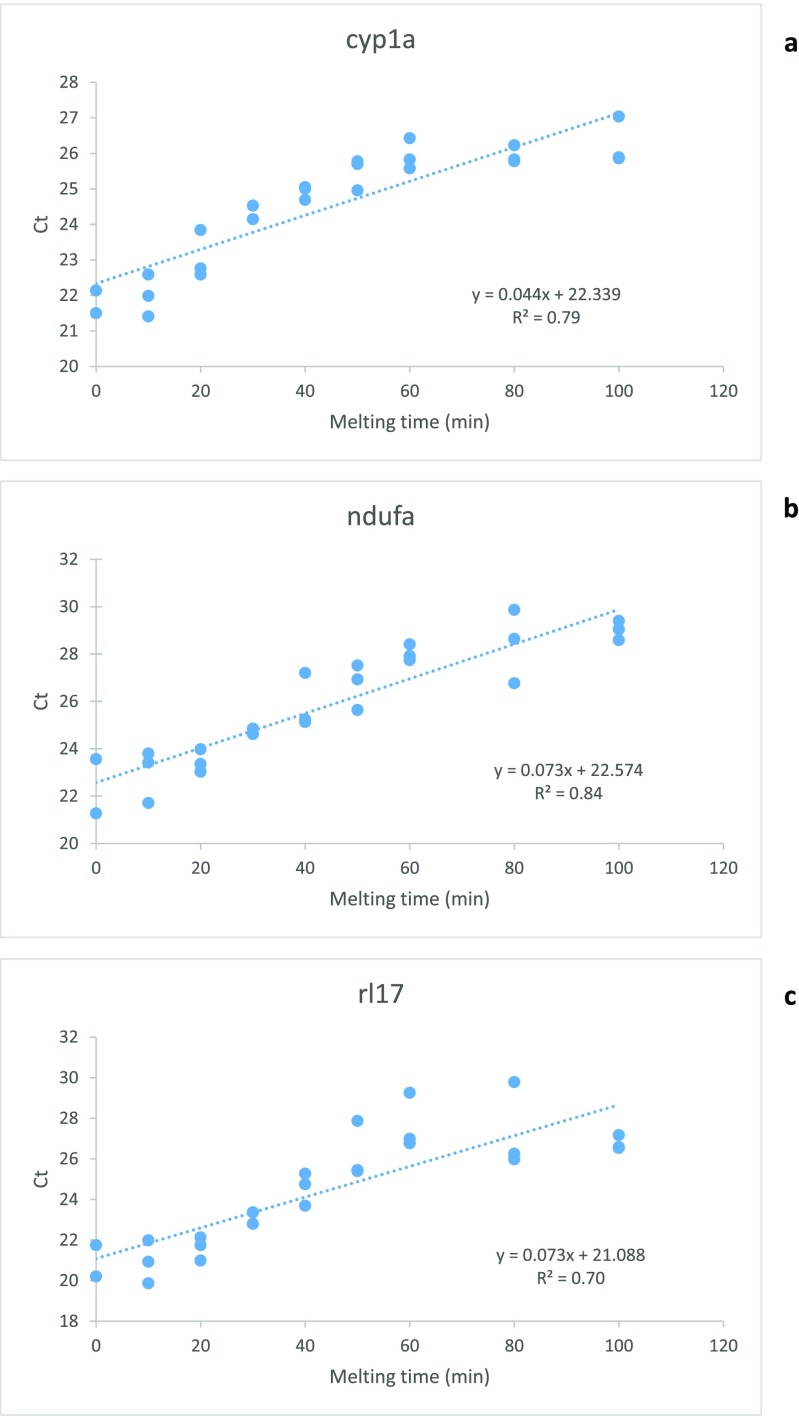
Melting of the samples affects the CT values. Three brown trout (*Salmo trutta*) livers that had been preserved in liquid nitrogen for 28 years were left to melt at room temperature, and samples collected at 0 to 100 min. RNA was extracted, reverse transcribed to cDNA, and qPCR was run with three genes. The figures present observed CT values plotted against melting time for the target gene cyp1a (**a**), ndufa (**b**), and rl17 (**c**) with their linear regression lines, their functions, and *R*^2^ values

**Fig. 4:**
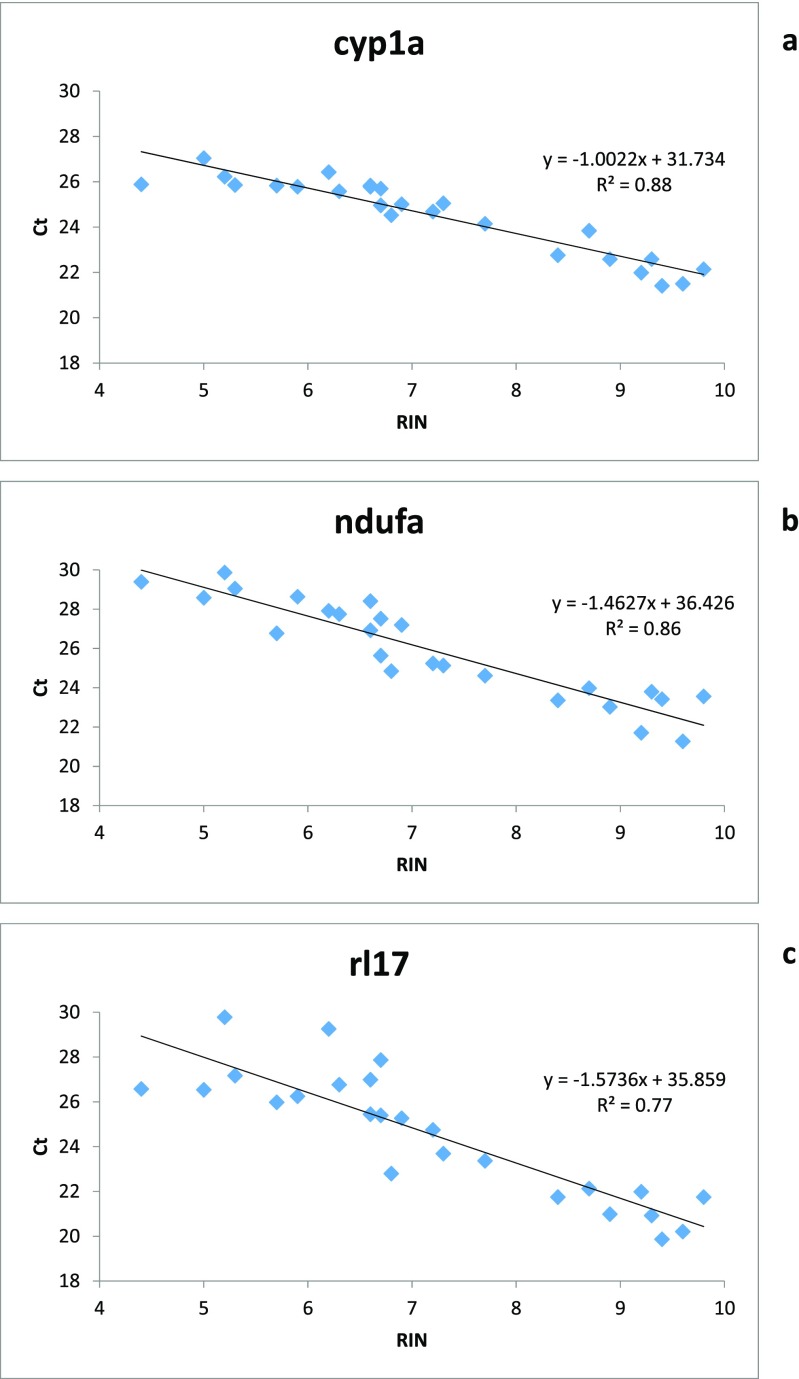
RNA degradation has an effect on CT values, and the effect is different between genes. Three brown trout (*Salmo trutta*) livers that had been preserved in liquid nitrogen for 28 years were left to melt at room temperature, and samples collected at 0 to 100 min to get identical samples with a varying degree of RNA degradation. RNA was extracted, reverse transcribed to cDNA, and qPCR was run with three genes. The figures present observed CT values plotted against RIN for the target gene cyp1a (**a**), ndufa (**b**), and rl17 (**c**) with their linear regression lines, their functions, and *R*^2^ values

Thawing the frozen liver tissue at room temperature caused a time-dependent deterioration in RNA integrity (repeated measures ANOVA with a Greenhouse-Geisser correction, *p* = 0.001) (Fig. 2). When qPCR was run for these samples, it was seen that the RNA degradation had an effect on C_T_ values and the effect was different between genes. The mRNA of the target gene *cyp1a* seemed to degrade more slowly than those of the reference genes, as the slopes of the linear regression curves of C_T_ as a function of sample melting time were different between *cyp1a* and *ndufa* (ANCOVA, *p* = 0.014) and *cyp1a* and *rl17* (ANCOVA, *p* = 0.018) (Fig. 3, Table 4). The slopes of the reference genes did not differ from each other (ANCOVA, *p* = 0.963). The more degraded the RNA (the lower the RIN) was, the higher the C_T_ value of every gene, which means that less target was present in the samples with a lower RIN (Fig. 4).

**Table 4:** Slopes of the linear regression lines of C_T_ values plotted against melting time of the liver sample and their 95% confidence intervals for target (*cyp1a*) and reference (*ndufa*, *rl17*) genes. The slopes of genes denoted with the same letter (a, b) do not differ statistically from each other (ANCOVA, *p* > 0.05)

Gene	Slope B, (95% confidence intervals)
*cyp1a*	0.044 (0.025,0.063) a
*ndufa*	0.073 (0.059, 0.087) b
*rl17*	0.073 (0.055, 0.090) b

### Similar *cyp1a* expression in all treatments in the historical samples, but significantly lower in new samples

**Fig. 5:**
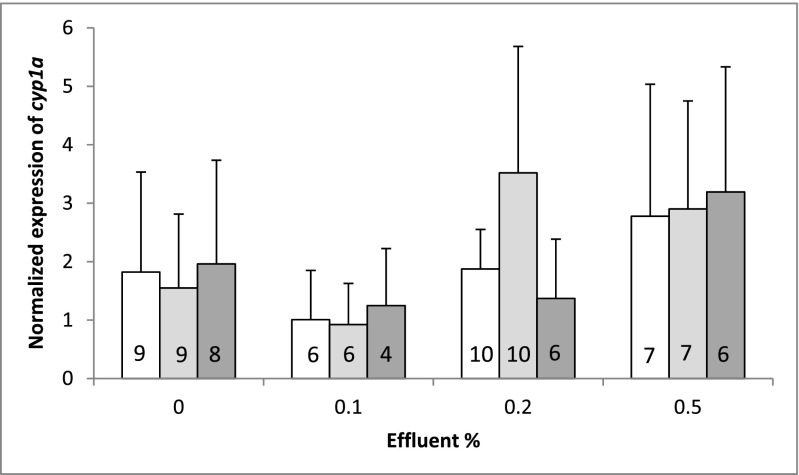
*cyp1a* gene expression in the liver of brown trout (*Salmo trutta*) exposed to dilutions of pulp and paper mill effluents for 4 months in 1984. The white columns represent measured expression values of samples with RIN ≥ 6, the light gray ones represent expression values calculated from RIN-corrected CT values of samples with RIN ≥ 6, and the dark gray ones represent measured expression values of samples with RIN 6–7. Data is presented as mean + SD. Number of analyzed samples is shown inside the columns

The *cyp1a* expression did not differ between treatments in the historical liver samples of brown trout (Fig. 5). Using samples with similar RINs (6–7) did not markedly change the results (no changes in statistical significance) (Fig. 5).The *cyp1a* mRNA levels were an order of magnitude higher in the historical control samples of unexposed brown trout than the new ones (Table 5). The magnitude of difference depended on the reference genes used, because the C_T_ values of *rl17* were remarkably higher in the historical samples than the new ones. While the *cyp1a* expression was almost 30 times higher in the historical than the new samples when both *ndufa* and *rl17* were used as reference genes, it was only 12 times higher when *ndufa* was the only reference gene (Table 5).

**Table 5:** Normalized expression of *cyp1a* in control brown trout from the 1984 study, and in brown trout of the same sex and approximately same age, sacrificed in 2012. Data is expressed as mean ± SD. *N* = 3 in both groups

	*cyp1a* expression, reference genes *ndufa* and *rl17*	*cyp1a* expression, reference gene *ndufa*
Historical control samples	0.86 ± 0.38	1.4 ± 0.7
New samples	0.03 ± 0.01	0.12 ± 0.07

## Discussion

### Effect of storage conditions

The higher the storage temperature and the longer the time of storage, the more degraded the liver RNA of brown trout was. In this study, the RNA had nearly completely degraded in samples left at room temperature for 24 h (mean RIN < 3). Preservation of samples at + 4 °C caused significant RNA degradation in 24 h (mean RIN < 7) and nearly complete loss of integrity in 5 days (mean RIN < 4). Previous studies have shown that it may be possible to extract good-quality RNA from human and rat tissues kept at room temperature for several hours after surgical removal (Bao et al. 2013; Micke et al. 2006; Ohashi et al. 2004; Thompson et al. 2007). However, controversial results have also been obtained, for example, 45-min room temperature preservation was enough to cause substantially more RNA degradation than ≤ 30 min (Viana et al. 2013). There are also observations that the vulnerability to RNA degradation during storage at room temperature may depend on tissue in both poikilotherms and homeotherms (Ibberson et al. 2009; Seear and Sweeney 2008; Viana et al. 2013). RNA may degrade very rapidly in tissues with high RNase activity, such as the pancreas, spleen, and lung—especially if there is any tissue damage so that the RNases stored in intracellular vesicles are released (Chirgwin et al. 1979). Our results with liver are applicable to tissues with low RNase content, such as the kidney, heart, and brain. As routine precaution preclude, it is advisable to avoid storing the samples at room temperature and in the fridge if good-quality RNA is needed.

Unexpectedly, no information was found in the published literature about the effect of − 20 °C storage on RNA integrity. Surprisingly, the RNA extracted from the liver stored at − 20 °C for 6 months was still rather intact (RIN ≥ 7). A normal freezer may thus be suitable for short-time preservation if a deep freezer is not available. Long-time preservation of samples at − 80 °C is recommended, as other studies have found that RNA integrity is not compromised by several years of storage at − 80 °C (Andreasson et al. 2013; Bao et al. 2013; Mathieson et al. 2016; Rudloff et al. 2010).

### Freeze-thaw cycles

Neither the freeze-thaw cycles of tissue nor the freeze-thaw cycles of purified RNA affected the integrity of RNA in brown trout liver samples. Previous studies on homeotherms have shown that freeze-thaw cycles themselves do not degrade RNA, but it is the time the RNA remains unfrozen that plays a role (Botling et al. 2009; Jochumsen et al. 2007; Thompson et al. 2007). The same seems to hold true for poikilotherms as well. A study with biobanked human half brains showed that the more times the brain had been sampled, the more degraded the RNA was (Sherwood et al. 2011). Though this result may seem controversial to ours, it was probably not the physical freeze-thawing that caused the degradation, as it can be speculated that the more times the brain had been sampled the longer it had remained unfrozen. Our results are applicable to samples that have intact RNA at the start of the preservation. Freeze-thaw cycles of low-quality samples may lead to more RNA degradation, as the RNases may have been released from cells before initial freezing. Based on our results, it can be concluded that both the samples and the extracted RNA may be thawed and frozen at least five times without compromising the RNA integrity, if the samples are of good quality (have been freshly frozen) and if the samples remain unfrozen on ice only for a short time (e.g., 30 min for tissue samples with low RNase content, 150 min for extracted RNA).

### RNA degradation may affect RT-qPCR results

In our study with a poikilothermic animal, RIN correlated well with C_T_ values: the lower the RIN value was, the higher the C_T_ value. This means signal detection at a later cycle, corresponding to less target sequence in the sample. Also, previous studies have shown that RNA degradation correlates with C_T_ values (Botling et al. 2009; Fleige et al. 2006; Koppelkamm et al. 2011; Perez-Novo et al. 2005; Vermeulen et al. 2011). However, RIN is greatly influenced by the degradation of ribosomal RNA, which does not always correlate well with the degradation of mRNA. For example, the correlation between RIN and C_T_ is poor in formalin-fixed paraffin-embedded samples (Kashofer et al. 2013). In mammalian samples, warm ischemia after removal of the tissue may have large effects on gene expression (Huang et al. 2001). This means that the C_T_ values may increase or decrease depending on the gene, and there is no correlation between C_T_ and RIN of the samples (Huang et al. 2001). In the present study, the fish were taken from cold water and the sampling procedure was performed in a low-temperature room. It is, however, of great importance to minimize the time between tissue removal and flash freezing.

The room temperature incubation affected the target gene *cyp1a* less than the two reference genes *ndufa* and *rl17*, which may mean that the mRNA of *cyp1a* degraded more slowly than those of the reference genes. These results are in agreement with earlier research showing that the magnitude of the effect of RNA degradation on C_T_ values may depend on gene (Botling et al. 2009; Fleige et al. 2006; Huang et al. 2013; Koppelkamm et al. 2011; Perez-Novo et al. 2005; Skrypina et al. 2003; Vermeulen et al. 2011). As the C_T_ value of *cyp1a* increases slower than the C_T_ values of the two reference genes when the RNA degrades, *cyp1a* expression will be overestimated in samples with low RIN values. It has been noticed that mammalian *cyp1a* mRNA may be extremely resistant to degradation (Skrypina et al. 2003). When oligo dT primers are used in the RT reaction, amplicons closer to 3′ end tend to degrade faster (Skrypina et al. 2003). In our work, primers were a mixture of oligo dT and random primers, and therefore the location of the amplicon respective to 3′ end may play a role. Another possible reason for the seemingly different degradation rate is that the *ndufa* and *rl17* amplicons are longer than the *cyp1a* amplicon. Moreover, there are observations that RNases may prefer certain sequences over others when they degrade RNA—even the most abundant form, RNase A (Boix et al. 2013; Wang et al. 2008). To avoid the possible biasing effect of differences created by RNA degradation, it is best to use samples with as intact RNA as possible, and with similar RINs, in transcriptomic analyses.

### *cyp1a* expression in historical and new samples

The historical samples of the control group fish showed remarkably higher *cyp1a* levels than the new ones. Though some of this difference could be explained by higher levels of the reference gene *rl17* in the new samples, there was still a difference by an order of magnitude when only *ndufa* was used as a reference gene. The historical and new fish were all of approximately the same age, but they originated from different stocks, which may play a role in the difference. However, a more likely explanation is the feed of the fish: The historical samples were from fish that, in addition to dry fish feed, had been fed with Baltic herring (*Clupea harengus* L.) (Vuorinen and Vuorinen 1985). At the time of the study, the Baltic herring contained significant amounts of dioxins and other persistent aryl hydrocarbon receptor (AhR) activators as can be judged from the total PCB concentrations in Baltic salmon, which prey on herring, from the 1980s to 2000s, 6.7 and 1.8 mg kg^−1^ (in fat), respectively (Vuorinen et al. 1985; Vuorinen et al. 2012).

Induction of CYP1A mRNA, protein, and activity levels is a biomarker of exposure to BKME in fishes (Gagnon et al. 1994; Kloepper-Sams and Benton 1994; Munkittrick et al. 1992; Soimasuo et al. 1995). Though a trend for increasing expression of *cyp1a* with increasing BKME concentration was seen in the historical samples investigated in this study, there was no statistically significant difference between treatments. Thus, one can conclude that either the effluent did not contain effective amounts of AhR activators, or the high basal *cyp1a* level (as seen in the control group fish) masked the effect of the effluent. Another possibility is that the BKME effluent contained estrogen-like compounds that repressed *cyp1a* expression (Navas and Segner 2001; Stegeman et al. 1982). The recruitment of the fish was compromised at 0.2 and 0.5% BKME effluent dilutions, which may point to disturbance of the steroid hormone pathways (Vuorinen, P. J. and Vuorinen 1985).

### Recommendations for the preservation and handling of fish samples and RNA for transcriptomic analyses

Storage: Store tissue and RNA designated for transcriptomic analyses at − 80 °C. Short-time storage at − 20 °C may be adequate for samples with low RNase content if − 80 °C storage is not available.

Freeze-thaw cycles: Liver tissue and purified RNA can be thawed at least five times without significant degradation of RNA, if the tissue samples have been frozen immediately after sampling, and if the time that the tissue or RNA remains unfrozen is kept in minimum.

Transcriptomic analyses: It is important to treat all samples in a similar way during sampling and storage. As different transcripts may degrade in a different way, we recommend using only samples with minimal degradation (as high RIN values as possible), and with similar RIN values.

These recommendations apply to tissues with relatively low RNase activity, such as the liver, kidney, and brain. Tissues with high RNase content (spleen, pancreas, stomach) may need to be treated in a different way to obtain good-quality RNA.
